# The adult with chronic cough and cat allergy

**DOI:** 10.1186/2045-7022-3-S1-P31

**Published:** 2013-05-03

**Authors:** Esmeralda Shehu, Mehmet Hoxha, Albana Deliu, Dorisa Meta

**Affiliations:** 1 Regional Hospital of Durres, Allergy and Clinical Immunology, Albania; 2 UHC Tirane, Allergy and Clinical Immunology, Albania

## Case report

A 31-year old man, tenor, present with a history of chronic cough of two years duration. The cough was present day and night, much more during singing, with little mucus production and some associated feeling of shortness of breath and choking sensation, especially at night. Additional complaints included postnasal drip, runny nose, sensation of mucus in the throat and symptoms of heartburn and acid indigestion. The patient was referred in our clinic, after inadequate response to previous treatments prescribed by previous specialists, including antihistamines, nasal steroid sprays, oral prednisone, antibiotics, combine therapy and proton-pump inhibitors. Physical examination was unremarkable. Spirometric values were normal. Results of chest radiography, computed tomographic scans of the sinuses, and high-resolution computed tomographic scans of the chest were all normal. The patient underwent a methacholine inhalation challenge. FEV1 was reduced 7% at the highest concentration (25 mg/ml). Bronchoscopy was normal . The esophageal pH monitoring was resulted positive. Skin prick test and RASTs resulted positive for cat dander. As the methacoline test was negative, patient was challenged in Italy, with a well-characterized cat-exposure model. Challenge result was considered positive for the upper respiratory symptoms, but there was no significant decline in FEV1 at 6 or 23 hours after cat exposure, the maximum fall was 9%. The patient was treated with cat-immunotherapy 15 μg of Fel d 1 at maintenance dose, and conservative therapy with combine therapy for 1 year, with no clinical improvement. He performed surgical intervention of the Nissen fundoplication, laparoscopically. There was significant improvement in quality of life only at 3 months after surgery. Because the patient's symptoms did not improve, we advised him to continue cat- immunotherapy, nasal steroid sprays, treatment with combine therapy and to stay away from the work and home environment for a period of time. Some months later the symptoms were significantly improved. Cat and dog allergens are commonly present in homes without pets, as well as in a variety of public buildings, and these are carried from one home to another on the clothing of pet owners. Non-cat owners can collect cat allergen on their clothing, most likely by both airborne and direct contact. Repeated low-level allergen exposure may induce chronic airway changes, specifically increases in airway hyperresponsiveness.

